# High post‐chemotherapy TIL and increased CD4+TIL are independent prognostic factors of surgically resected NSCLC following neoadjuvant chemotherapy

**DOI:** 10.1002/mco2.213

**Published:** 2023-02-09

**Authors:** Wenxiao Jia, Hongbo Guo, Min Wang, Ji Li, Jinming Yu, Hui Zhu, Gang Wu

**Affiliations:** ^1^ Cancer Center, Union Hospital, Tongji Medical College Huazhong University of Science and Technology Wuhan Hubei China; ^2^ Department of Radiation Oncology, Shandong Cancer Hospital and Institute, Shandong First Medical University Shandong Academy of Medical Sciences Jinan Shandong China; ^3^ Department of Thoracic Surgery, Shandong Cancer Hospital and Institute, Shandong First Medical University Shandong Academy of Medical Sciences Jinan Shandong China

**Keywords:** CD4, CD8, immunotherapy, neoadjuvant chemotherapy, NSCLC, tumor‐infiltrating lymphocyte

## Abstract

Neoadjuvant chemotherapy (NCT) has significantly improved the overall survival of patients with operable non‐small cell lung cancer (NSCLC). Chemotherapy can remodel the tumor immune microenvironment (TIME) and has an important influence on antitumor immunity. For patients who underwent surgery for resected NSCLC following NCT (NCT‐NSCLC), a prognostic value comparison between naïve and post‐chemotherapy TIME is absent. We enrolled 89 patients with NCT‐NSCLC in this study; the tumor‐infiltrating lymphocyte (TIL), CD4+TIL, and CD8+TIL levels in naïve and post‐chemotherapy tumor tissues were detected using immunohistochemistry staining and divided into high and low groups. Kaplan–Meier analysis revealed that major pathology response, pathological tumor, node, and metastasis stage post‐NCT (ypTNM), high post‐chemotherapy TIL, high post‐chemotherapy CD8+TIL, low naïve CD4+TIL, low naïve CD4+/CD8+TIL ratio, and increased CD4+TIL levels post‐chemotherapy were favorable prognostic factors in patients with NCT‐NSCLC. Multivariate Cox analysis found that ypTNM, high post‐chemotherapy TIL, and increased CD4+TIL levels post‐chemotherapy were independent prognostic factors in patients with NCT‐NSCLC. These results indicate that a TIME remodeled by chemotherapy plays an important role in antitumor immunity and has a better prognostic value than the naïve TIME.

## Graphical abstract

NCT remodeled the infiltrating level of CD4+TIL and CD8+TIL in individuals, which is important for antitumor immunity and associated with the prognosis of NCT‐NSCLC. The ypTNM stage, post‐chemotherapy TIL, and CD4+TIL ascend post‐chemotherapy is independent prognostic factors of NCT‐NSCLC. This study indicated that TIME remodeled by chemotherapy has a better prognostic value than the naïve TIME.

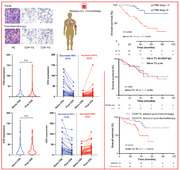

## 1 INTRODUCTION

Lung cancer is the leading cause of cancer‐related deaths worldwide, with over 85% of these cases classified as non‐small cell lung cancer (NSCLC).^1^ Surgery is the main radical treatment for NSCLC; however, direct surgery is difficult in some patients with stage III NSCLC, especially the N2 stage. Neoadjuvant chemotherapy (NCT) (no less than two cycles of chemotherapy before surgery) was delivered to these patients to reduce the tumor stage, improve operability, and eliminate micro‐metastatic disease.^2^, ^3^ In 2014, the NSCLC Meta‐Analysis Collaborative Group reported that NCT can improve the 5‐year overall survival (OS) rate from 40% to 45% for stage IB–IIIA NSCLC.^4^ The CSLC 0501 study suggested that NCT significantly improved the OS compared with adjuvant chemotherapy, with 5‐year OS rates of 42.1% and 57.8%, respectively.^5^ A previous study suggested that NCT is associated with prognosis in patients with NSCLC and can remodel the tumor immune microenvironment (TIME), which is important in tumor development.^6^, ^7^

In 2022, Park et al.^8^ discovered that different tumor‐infiltrating lymphocyte (TIL) phenotypes correlated with the tumor response to immune checkpoint inhibitors, and this is similar to the results of Shirasawa et al. in 2021.^9^ However, in 2012, Liu et al.^10^ found that in advanced NSCLC, naïve TIL levels did not correlate with tumor response to chemotherapy. CD4+ T and CD8+ T cells constitute the main components of the TIL and play different roles in antitumor immunity; they are also composed of different cell subtypes.^11^, ^12^, ^13^, ^14^ In general, high CD8+ T‐cell infiltration is a favorable prognostic factor for NSCLC.^15^, ^16^, ^17^ However, CD4+ T cells have high functional heterogeneity and have been reported to have different prognostic values in NSCLC.^11^, ^14^, ^18^

Gaudreau et al.^6^ found that NCT can remodel the TIME and increase cytotoxic T cells and tissue‐resident memory T‐cell infiltration. Parra et al.^7^ also found that patients with NSCLC who received NCT followed by surgery (NCT‐NSCLC) had higher infiltrating levels of epithelial CD3+CD4+ T lymphocytes and CD68+ epithelial and stromal tumor‐associated macrophages than patients who underwent upfront surgery (non‐NCT‐NSCLC). However, the naïve and post‐chemotherapy tumor tissue specimens for these studies were obtained from different patients.

In this study, we examined the infiltrating level of TIL, CD4+TIL, and CD8+TIL in naïve and post‐chemotherapy NCT‐NSCLC tumor tissue specimens, and explored their prognostic value in patients with NCT‐NSCLC, both naïve and post‐chemotherapy tumor tissue specimens came from the same patients. We found that major pathology response (MPR), pathological tumor, node, and metastasis stage post‐NCT (ypTNM), post‐chemotherapy TIL, post‐chemotherapy CD8+TIL, naïve CD4+TIL, naïve CD4+/CD8+TIL ratio, and change of CD4+TIL are prognostic factors of NCT‐NSCLC. Among them, ypTNM, high post‐chemotherapy TIL, and increased CD4+TIL levels post‐chemotherapy were independent prognostic factors in patients with NCT‐NSCLC.

## 2 RESULT

### 2.1 Patient characteristics

**FIGURE 1:**
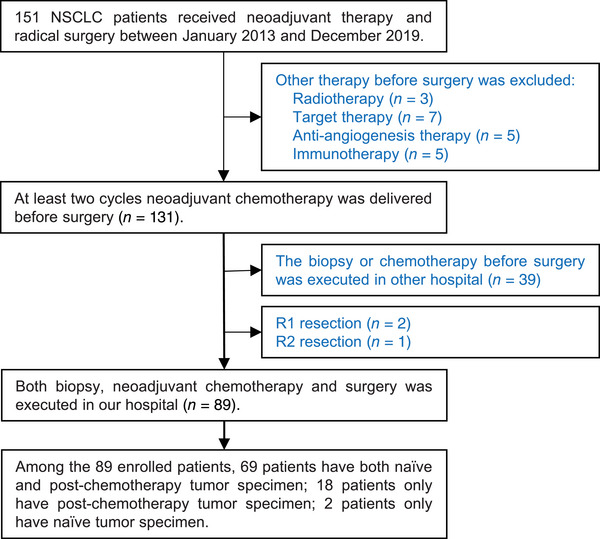
The flowchart for patient recruitment

All patients with surgically resected NSCLC at Shandong Cancer Hospital and Institute were screened from January 2013 to December 2019, and 89 patients were enrolled according to the inclusion and exclusion criteria. The patient screen flowchart is presented in Figure 1. Among them, 70.79% of the patients were male with a median age of 64 years, and main histopathological types were adenocarcinoma and squamous carcinoma. The median follow‐up time was 39.80 months and the median OS was 70.30 months. The distributions of clinical tumor, node, and metastasis (cTNM) stages I, II, and III were 12 (13.48%), 10 (11.24%), and 67 (75.28%), respectively. The NCT regimens were platinum combined with pemetrexed, paclitaxel, or gemcitabine, and all patients received R0 resections. Following NCT, 30 patients experienced descending TNM stages, and 10 patients experienced ascending TNM stages. Among the patients with ascending TNM stages, six had an elevated N stage, and four had an elevated T stage. The distributions of ypTNM stages I, II, and III were 25 (28.09%), 19 (21.35%), and 45 (50.56%), respectively. Following NCT, 10 patients achieved MPR; among them, six patients achieved a complete pathological response. The clinicopathological characteristics of all patients are presented in Table 1.

**TABLE 1:** Clinicodemographic characteristics of all patients

Factor	*N* (%)
Gender (*n*)	
Male	63 (70.79)
Female	26 (29.21)
Age (years), median (range)	64 (36–81)
Smoking history (*n*)	
No	37 (41.57)
Yes	52 (58.43)
Histopathology	
Adenocarcinoma	50 (56.18)
EGFR mutation	11 (22.00)
ALK rearrangement	2 (4)
ROS1 rearrangement	2 (4)
Driver gene negative	18 (36)
Not available	17 (34)
Squamous	37 (41.57)
Adenosquamous carcinoma	2 (2.25)
cTNM stage (*n*)	
I	12 (13.48)
II	10 (11.24)
III	67 (75.28)
ypTNM stage (*n*)	
I	25 (28.09)
II	19 (21.35)
III	45 (50.56)
Neoadjuvant chemotherapy regimen	
Pemetrexed + platinum	40 (44.94)
Paclitaxel + platinum	20 (23.59)
Gemcitabine + platinum	29 (32.58)
Pathology response	
MPR	79 (88.76)
Non‐MPR	10 (11.24)
Naïve TIL	
Low	61 (68.54)
Median	18 (20.22)
High	2 (2.25)
Post‐chemotherapy TIL	
Low	25 (28.09)
Median	50 (56.18)
High	14 (15.73%)
Naïve CD4 *H*‐score, median (range)	7.94 (0–132)
Post‐chemotherapy CD4 *H*‐score, median (range)	8.64 (0.72–151.21)
Naïve CD8 *H*‐score, median (range)	16.05 (1–167)
Post‐chemotherapy CD8 *H*‐score, median (range)	16.19 (0.45–120.51)

Abbreviations: ALK, anaplastic lymphoma kinase; cTNM, clinical tumor, node, and metastasis; EGFR, epidermal growth factor receptor; MPR, major pathology response; ROS1, proto‐oncogene receptor tyrosine kinase; TIL, tumor‐infiltrating lymphocytes; TKI, tyrosine kinase inhibitor; ypTNM, pathological TNM stage post‐neoadjuvant chemotherapy.

### 2.2 NCT increased post‐chemotherapy TIL and remodeled the infiltration of CD4+TIL and CD8+TIL in individuals

**FIGURE 2:**
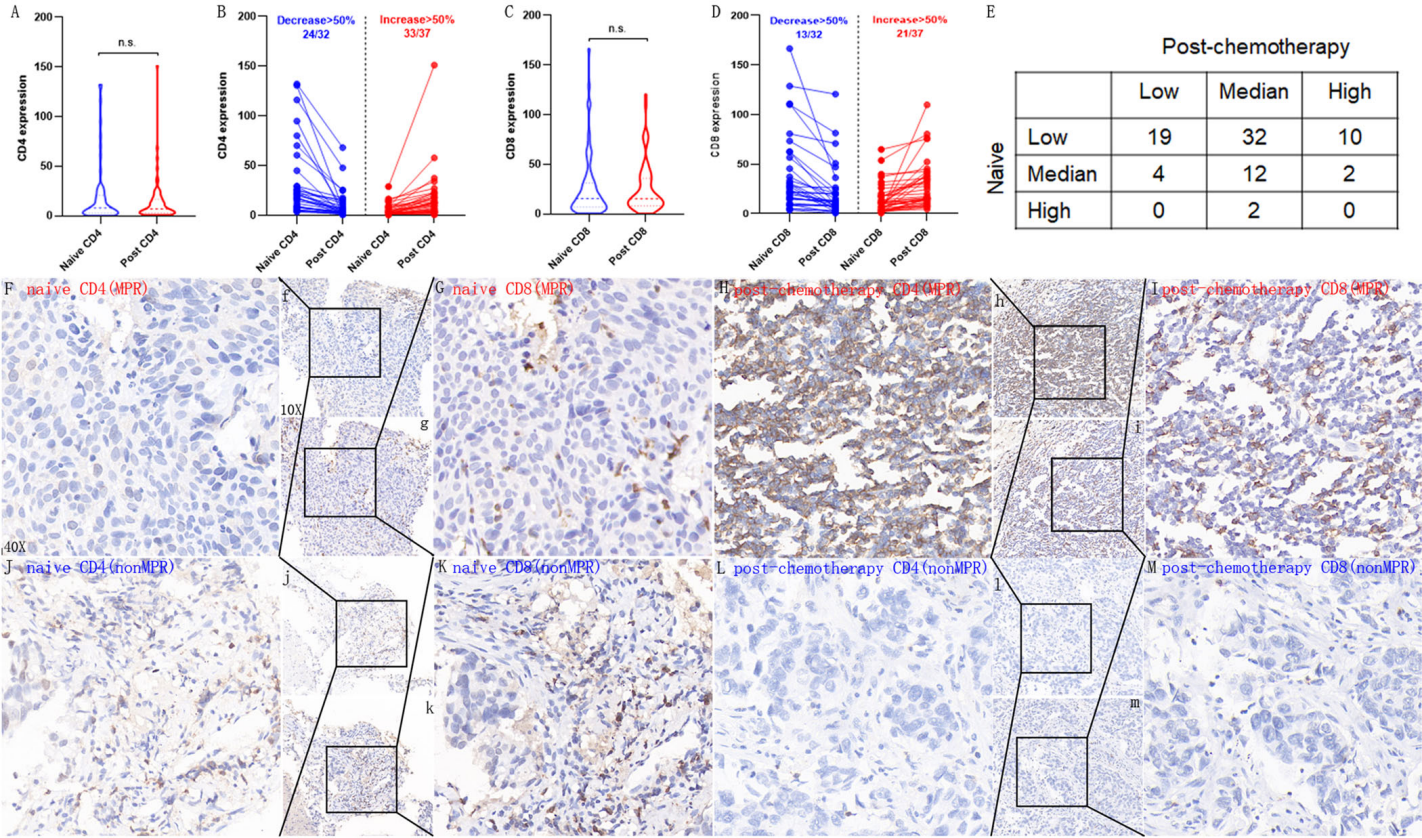
(A–E) The change in CD4+TIL, CD8+TIL, and total tumor‐infiltrating lymphocytes (TIL) following neoadjuvant chemotherapy (NCT). (F–H) The represented immunohistochemistry staining results of two patients who achieved a complete pathological response and non‐major pathology response (MPR) post‐NCT. No difference was observed in naïve and post‐chemotherapy CD4+TIL or CD8+TIL in all the patients (A and C); 75% and 40.63% of patients have >50% decreased CD4+TIL and CD8+TIL infiltrating, respectively; 89.19% and 56.76% of patients have >50% increased CD4+TIL and CD8+TIL, respectively (B and D). Forty‐four and six patients had increased or decreased total TIL infiltration, respectively (E). The represented 10× and 40× images of patient CD4 and CD8 expressions achieved complete pathological response in naïve tumor (F, f and G, g) and post‐chemotherapy tumor specimen (H, h and I, i). The represented 10× and 40× images of CD4 expression and CD8 expression of non‐MPR patient in naïve tumor (J, j and K, k) and post‐chemotherapy tumor specimen (L, l and M, m).

The distributions of naïve TIL were 61 (68.54%), 18 (20.22%), and 2 (2.25%) for the low, middle, and high groups, respectively. Among 81 patients with paired naïve and post‐chemotherapy TIL result, six patients had decreased TIL infiltration, and 44 had increased TIL infiltration (Figure 2E); NCT significantly increased the TIL infiltration in patients with NCT‐NSCLC. The post‐chemotherapy distributions for the low, middle, and high were 25 (28.09%), 50 (56.18%), and 14 (15.73%), respectively. When switched to the important component of TIL: CD4+TIL and CD8+TIL, we found no difference between naïve and post‐chemotherapy infiltrating levels for both CD4+TIL and CD8+TIL in all the patients (Figure 2A,C). The median naïve and post‐chemotherapy CD4 *H*‐scores were 7.94 and 8.64, respectively, and those for CD8, *H*‐scores were 16.05 and 16.19, respectively. When focused on the individual patients, however, we found that 82.61% (57/69) and 49.28% (34/69) had >50% decreased or increased CD4+TIL and CD8+TIL compared with naïve infiltrating levels post‐NCT, respectively (Figure 2B,D). Therefore, we concluded that NCT remodeled the CD4+TIL and CD8+TIL infiltration in individuals, which was not found when all the patients were analyzed. Representative CD4 and CD8 immunohistochemical staining results of two patients who had significant increase or decrease following NCT are presented in Figure 2F–M. One of them achieved complete pathological response and the other did not achieve MPR.

### 2.3 ypTNM, high post‐chemotherapy TIL, and MPR are favorable prognostic factors for NCT‐NSCLC

**FIGURE 3:**
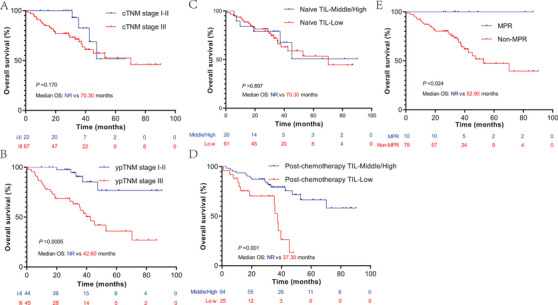
Kaplan–Meier survival analysis of clinical TNM (cTNM) (A), pathological TNM stage post‐neoadjuvant chemotherapy (ypTNM) (B), tumor‐infiltrating lymphocytes (TIL) infiltration level of naïve (C) and post‐chemotherapy (D) tumor tissue, and major pathology response (MPR) (E) for non‐small cell lung cancer following neoadjuvant chemotherapy (NCT‐NSCLC).

The cTNM, ypTNM, MPR, naïve, and post‐chemotherapy TIL were analyzed to evaluate their prognostic value in patients with NCT‐NSCLC. As expected, we found that patients with ypTNM stage III had worse OS rates than those with ypTNM stage I–II (*p* = 0.0005, median OS: 42.60 months vs. not reached [NR]), but this phenomenon was not observed in cTNM (Figure 3A,B). Similarly, we also found that the post‐chemotherapy TIL infiltration level was a prognostic factor of OS, rather than the naïve TIL infiltration level. Patients with middle or high post‐chemotherapy TIL level had significantly longer OS than those with low TIL infiltration levels (*p* = 0.001, median OS: NR vs. 37.30 months) (Figure 3C,D). In addition, we found that patients who achieved MPR had a significant survival benefit compared to those who did not (*p* = 0.024, median OS: NR vs. 52.90 months) (Figure 3E). In this section, we found that ypTNM, high post‐chemotherapy TIL, and MPR are favorable prognostic factors for NCT‐NSCLC.

### 2.4 High naïve CD4+TIL and low post‐chemotherapy CD8+TIL are associated with poor OS of NCT‐NSCLC

**FIGURE 4:**
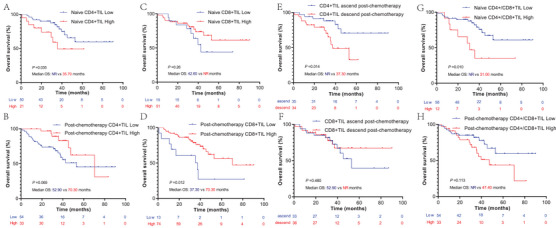
(A–H) Kaplan–Meier survival analysis of CD4+TIL and CD8+TIL infiltrates, CD4+/CD8+TIL ratio in naïve and post‐chemotherapy tumor tissues, and CD4+TIL and CD8+TIL change post‐chemotherapy for non‐small cell lung cancer following neoadjuvant chemotherapy (NCT‐NSCLC). TIL, tumor‐infiltrating lymphocytes

The cutoff values of naïve, post‐chemotherapy CD4, and CD8 *H*‐scores were decided by an X‐tile based on the OS. According to the cutoff value, these patients were divided into CD4+TIL low or high groups and CD8+TIL low or high groups. Kaplan–Meier survival analysis found that patients with low naïve CD4+TIL had a significant survival benefit compared to those with high naïve CD4+TIL (*p* = 0.035, median OS: NR vs. 35.70 months) (Figure 4A). However, when focusing on the post‐chemotherapy CD4+TIL group, we observed a contradictory result: the high post‐chemotherapy CD4+TIL group presented an OS benefit trend compared to the low post‐chemotherapy CD4+TIL group (*p* = 0.069) (Figure 4B). A high post‐chemotherapy CD8+TIL was also predicted to have a longer survival than low post‐chemotherapy CD8+TIL (*p* = 0.012, median OS: 37.30 vs. 70.30 months), and this difference was not observed in naïve CD8+TIL (Figure 4C,D). In this section, we found that high naïve CD4+TIL and low post‐chemotherapy CD8+TIL are associated with poor OS of NCT‐NSCLC.

### 2.5 CD4+TIL ascend post‐chemotherapy and low naïve CD4+/CD8+TIL ratio are favorable prognostic factors for NCT‐NSCLC

To better explore the prognostic value of CD4+TIL and CD8+TIL in patients with NCT‐NSCLC, the CD4+/CD8+TIL ratio and change in CD4+TIL and CD8+TIL levels were analyzed using Kaplan–Meier survival analysis. According to this result, we found that patients with ascend CD4+TIL levels post‐chemotherapy had a significant survival benefit compared to those with decreased CD4+TIL levels (*p* = 0.024, median OS: NR vs. 37.30 months) (Figure 4E). No survival difference was observed in patients with ascending or descending CD8+TIL post‐chemotherapy (Figure 4F). Patients with a low naïve CD4+/CD8+TIL ratio also had a longer median OS than patients with a high naïve CD4+/CD8+TIL ratio (*p* = 0.010, median OS: NR vs. 31.00 months), and this phenomenon was not observed for the post‐chemotherapy CD4+/CD8+TIL ratio (Figure 4G,H). In this section, we found that is CD4+TIL ascend post‐chemotherapy and low naïve CD4+/CD8+TIL ratio associated with favorable prognostic of NCT‐NSCLC.

### 2.6 ypTNM, post‐chemotherapy TIL, and CD4+TIL ascent are independent prognosis factors for NCT‐NSCLC

cTNM, ypTNM, pathological response, naïve and post‐chemotherapy TIL, naïve and post‐chemotherapy CD4+TIL, CD8+TIL, naïve and post‐chemotherapy CD4+/CD8+TIL ratio, and changes in CD4+TIL and CD8+TIL levels post‐chemotherapy were enrolled in univariate and multivariate Cox analyses. Consistent with the above results, univariate Cox analysis revealed that ypTNM, post‐chemotherapy TIL, naïve CD4+TIL, post‐chemotherapy CD8+TIL, change in CD4+TIL, and naïve CD4+/CD8+TIL ratio were prognostic factors of OS for NCT‐NSCLC (Table 2). In multivariate Cox analysis, we found that ypTNM (*p* = 0.033, hazard ratio [HR] = 3.48, 95% confidence interval [CI]: 1.11–10.94), post‐chemotherapy TIL (*p* = 0.014, HR = 0.29, 95% CI: 0.10–0.91), and an increase in CD4+TIL levels post‐chemotherapy (*p* = 0.019, HR = 0.29, 95% CI: 0.10–0.81) were independent prognostic factors of NCT‐NSCLC (Table 2). In view of the potential relationship between ypTNM and TIL, the prognostic value of post‐chemotherapy TIL and CD4+TIL change was analyzed in patients with ypTNM stage III NCT‐NSCLC. As we expected, the prognostic value of post‐chemotherapy TIL and CD4+TIL change is consistent in ypTNM stage III and all the patients, which confirmed that the prognostic value of post‐chemotherapy TIL and CD4+TIL is independent of ypTNM (Figure S1). These results indicated that TNM, TIL, and CD4+TIL have a higher prognostic value in post‐chemotherapy than in naïve tumor tissue specimens.

**TABLE 2:** Univariate and multivariate Cox analyses of the prognostic factors of overall survival

Factor	Univariate Cox analysis		Multivariate Cox analysis	
	HR (95% CI)	*p*‐Value	HR (95% CI)	*p*‐Value
cTNM stage				
I–II				
III	2.06 (0.72–5.93)	0.180		
ypTNM stage				
I–II				
III	**4.32 (1.76**–**10.58)**	**0.001**	**3.48 (1.11**–**10.94)**	**0.033**
Pathology response				
Non‐MPR				
MPR	0.039 (0.0005–3.28)	0.151		
Naïve TIL				
Low				
Median/high	1.06 (0.45–2.49)	0.90		
Post‐chemotherapy TIL				
Low				
Median/high	**0.29 (0.14**–**0.62)**	**0.001**	**0.29 (0.10**–**0.91)**	**0.014**
Naïve CD4 *H*‐score				
Low				
High	**2.45 (1.04**–**5.80)**	**0.041**		
Post‐chemotherapy CD4 *H*‐score				
Low				
High	0.46 (0.20–1.08)	0.076		
Naïve CD8 *H*‐score				
Low				
High	0.61 (0.25–1.47)	0.27		
Post‐chemotherapy CD8 *H*‐score				
Low				
High	**0.37 (0.16**–**0.83)**	**0.016**		
Change of CD4 *H*‐score				
Descend				
Ascend	**0.34 (0.13**–**0.84)**	**0.019**	**0.29 (0.10**–**0.81)**	**0.019**
Change of CD8 *H*‐score				
Descend				
Ascend	1.37 (0.57–3.25)	0.48		
Naïve CD4/CD8 ratio				
Low				
High	**3.13 (1.26**–**7.82)**	**0.014**		
Post‐chemotherapy CD4/CD8 ratio				
Low				
High	1.79 (0.86–3.710)	0.12		

Abbreviations: CI, confidence interval; cTNM, clinical TNM; HR, hazard ratio; MPR, major pathology response; TIL, tumor‐infiltrating lymphocytes; ypTNM, pathological TNM stage post‐neoadjuvant chemotherapy.

## 3 DISCUSSION

NCT has brought significant survival benefits to patients with surgically resected NSCLC.^4^, ^5^ Previous studies suggested that NCT plays an important role in antitumor immunity can remodel the TIME.^6^, ^7^ Gaudreau et al.^6^ found that NCT increases the infiltration of cytotoxic CD8+ T cells and CD20+ B cells compared with upfront surgery. Parra et al.^7^ found that patients who received NCT followed by surgery had a higher infiltration of epithelial CD3+CD4+ T lymphocytes and CD68+ epithelial and stromal tumor‐associated macrophages. Many studies have presented that CD4+ T cells are a high heterogeneous population and have different subtypes and functions.^11^, ^14^ Cohen et al.^19^ found that the CD4+ T‐cell subtype T‐helper cells can interact with dendritic cells and be primed in tumor‐draining lymph nodes, which is important for harnessing the antitumor immune response of anti‐PD‐1 treatment. Regulatory T cells are considered an immunosuppressive T‐cell subtype that suppress the antitumor immune response.^18^ In general, CD8+ T cells have showed effective antitumor immune responses except for some CD8+ T cell with exhausted phenotypes.^16^, ^17^, ^20^

Although these studies have reported the prognostic value of the TIME, most of them focused on advanced NSCLC cases, and the tumor tissue specimens were mainly derived from naïve tumor tissues. Other studies on the TIME of NCT‐NSCLC have mainly focused on the TIME differences between NCT‐NSCLC and non‐NCT‐NSCLC. The prognostic value of TIME in NCT‐NSCLC has received less attention, and a prognostic value comparison between naïve and post‐chemotherapy tumor tissue specimens in NCT‐NSCLC had never been reported. In this study, we assessed the total TIL, CD4+TIL, and CD8+TIL levels in naïve and post‐chemotherapy tumor tissue specimens from the same patients and explored the prognostic value differences between naïve and post‐chemotherapy tumor tissue specimens.

As expected, we found that NCT can significantly increase the TIL infiltration. However, we found no differences between naïve and post‐chemotherapy CD4+TIL or CD8+TIL when focused on all the patients. But, when individual CD4+TIL or CD8+TIL were analyzed, we found that NCT significantly remodeled the CD4+TIL and CD8+TIL in 82.61% and 49.28% of patients, respectively. This result reminded us that the immune remodeled response to chemotherapy is individual, and this phenomenon can be ignored without paired tumor specimens.

Similar to a previous study, we found that MPR and early ypTNM were favorable prognostic factors in patients with NCT‐NSCLC; however, this phenomenon was not observed in cTNM, which can be explained by the response difference to chemotherapy and inaccurate cTNM stage, especially for the regional lymph nodal stage.^21^ Although many studies have indicated that TIL infiltration is associated with tumor response to immune checkpoint inhibitors, when focusing on chemotherapy, the TIL level is not associated with the prognosis of patients with advanced NSCLC. Similar to this study, we also found that the naïve TIL level was not associated with the prognosis of NCT‐NSCLC.^8^, ^9^, ^10^ However, when switching to post‐chemotherapy TIL levels, unlike naïve TIL levels, we found that a high post‐chemotherapy TIL level significantly prolonged the OS of NCT‐NSCLC. This phenomenon indicated that the post‐chemotherapy TIL level, rather than naïve TIL, is a prognostic factor of NCT‐NSCLC. According to this result, we speculate that the TIME remodeled by NCT represented antitumor immune activity rather than the naïve TIME.

Additionally, we found that patients with NCT‐NSCLC with high naïve CD4+TIL levels had shorter OS than those with low naïve CD4+TIL levels, and an opposite phenomenon was observed post‐chemotherapy (*p* = 0.069). We also found that CD4+TIL increase post‐chemotherapy was a favorable prognostic factor. As previously reported, CD4+TIL is highly heterogeneous and has different immune functions.^11^, ^18^ According to this result, we speculate that naïve CD4+TIL may mainly constitute of immunosuppressed CD4+ T cells, and that highly immunosuppressed naïve CD4+TIL form the immunosuppressive TIME, which prevents activated CD4+TIL infiltration post‐chemotherapy. For patients with low naïve CD4+TIL, NCT may increase the infiltration of immune‐activated CD4+TIL, enhance antitumor immunity, and prolong the OS of patients with NCT‐NSCLC. When focusing on CD8+TIL, our result is consistent with the previous study that high post‐chemotherapy CD8+TIL represented a longer OS for patients with NCT‐NSCLC.^22^, ^23^, ^24^ The high naïve CD4+/CD8+TIL ratio also represented a poor prognostic factor in patients with NCT‐NSCLC, which was consistent with previous study on operations for NSCLC and gastric cancer.^25^, ^26^

In multivariate Cox analysis, we found that ypTNM, post‐chemotherapy TIL, and increased CD4+TIL levels post‐chemotherapy were independent prognostic factors of OS. This result was consistent with the Kaplan–Meier survival analysis, and all these results indicate that post‐chemotherapy TIME has a higher prognostic value than naïve TIME, indicating that TIME remodeled by chemotherapy plays an important role in antitumor immunity and exerts antitumor effects.

Recently, immunotherapy has been introduced as a neoadjuvant therapy for NSCLC and has significant MPR benefits compared with NCT.^27^, ^28^, ^29^ However, owing to the long‐term survival of surgically resected NSCLC after neoadjuvant immunotherapy, there are no mature survival data for neoadjuvant immunotherapy or immunochemotherapy in NSCLC.^30^, ^31^, ^32^ In our study, we found that CD4+TIL increases post‐chemotherapy, and high post‐chemotherapy TIL has a significant survival benefit, which suggests that the addition of immunotherapy may prolong the survival of patients with NSCLC. Furthermore, for some patients with high naïve CD4+TIL, NCT alone may be insufficient, and the addition of immunotherapy may improve the prognosis of these patients.

However, some limitations cannot be ignored in this study. Firstly, the naïve tumor tissue came from biopsy specimen, and owing to the spatial heterogeneity of TIME, the biopsy specimen may not perfectly represent the whole TIME. Secondly, many patients were excluded because the naïve TIL level could not be assessed. Only 89 patients with NCT‐NSCLC were enrolled in this study, and of this number, the naïve biopsy specimen could not be obtained for a few patients. Thirdly, there were 12 patients with stage I NSCLC who received NCT and were enrolled in this cohort, which is not suitable for NCT. In order to reflect the real clinical status, we still decided to enroll these patients in this study. Finally, in general, disease‐free survival is the primary endpoint for NCT‐NSCLC study, but in this retrospective study, we explored the prognostic value of TIL, which reflected the systemic antitumor immunity to some extent. In addition, the systemic antitumor immunity is believed to be an important prognostic factor of OS for patients with NSCLC, so we chose the OS as the study endpoint. Meanwhile, in order to exclude the influence of follow‐up treatment, we reanalyzed the prognostic value of various TIL after eliminating the patients who received subsequent epidermal growth factor receptor or anaplastic lymphoma kinase tyrosine kinase inhibitor treatment because of the important influence on OS, and the result was consistent with that of all patients (Figures S2 and S3), which also illustrated that the TIL is independent prognostic factor of OS for NSC‐NSCLC.

In this study, we found that ypTNM, high post‐chemotherapy TIL, and increased CD4+TIL levels post‐chemotherapy were independent prognostic factors in patients with NCT‐NSCLC. This result indicates that post‐chemotherapy TIME, rather than naïve TIME, plays an important antitumor role and predicts the prognosis of NCT‐NSCLC. The naïve TIME can be remodeled by NCT and alter antitumor immune effects. For some patients with high naïve CD4+TIL, NCT may have difficulty activating their TIME, and the addition of immunotherapy can be a better option to activate antitumor immunity and improve survival.

## 4 MATERIALS AND METHODS

This study was conducted in accordance with the Declaration of Helsinki. The study protocol was reviewed and approved by the institutional review board and ethics committee of Shandong Cancer Hospital and Institute (ethical number: SDTHEC2021001025). All patients provided informed consent before paraffin‐embedded tissue sections were obtained. The medical records were reviewed using an institutional query system.

### 4.1 Patients

All NSCLC cases surgically resected post‐NCT at Shandong Cancer Hospital and Institute between January 2013 and December 2019 were retrieved. The inclusion criteria were as follows: (1) stage I–III NSCLC; (2) a biopsy for diagnosis and surgery performed in our hospital; and (3) paraffin‐embedded tissue sections reserved in our hospital. Informed consent was obtained from patients or authorized persons. The exclusion criteria were as follows: (1) small cell lung cancer; (2) R1 or R2 resection; and (3) other treatments before surgery, such as radiotherapy, targeted therapy, anti‐angiogenesis therapy, and immunotherapy. All patients received at least two cycles of NCT and radical surgery. The chemotherapy regimens were combined platinum and pemetrexed, paclitaxel, or gemcitabine. The surgical approaches included thoracotomy and video‐assisted thoracoscopic surgery. cTNM and ypTNM were restaged according to AJCC Cancer Staging (8th edition).

### 4.2 Tumor infiltration lymphocyte and MPR assessment

At least three hematoxylin–eosin sections at diagnosis were used to assess the TIL infiltration level. The residual tumor cell rate was independently assessed by at least two senior pathologists, with a third senior pathologist involved in cases with inconsistent results. The TIL infiltration levels were classified as low, middle, or high using the IASLC Neoadjuvant Pathology Recommendations.^33^ The middle and high TIL levels were classified as one group for subsequent survival analysis. The residual tumor cells were assessed and recorded from 0% to 100% at 10% intervals, and the MPR was defined as the residual tumor cell in the tumor bed from 0% to 10%.^34^, ^35^

### 4.3 Immunohistochemistry stain and immunostaining scoring analysis

Paraffin‐embedded tumor tissues were cut into 4 μm serial slides and dried at 60°C for 1 h. Tissue slides were deparaffinized in xylene, rehydrated with an alcohol gradient, and washed with purified water. Antigen retrieval was performed using citrate buffer (pH 6.0) at 95°C for 20 min. After heating, the slides were cooled to room temperature and washed in phosphate‐buffered saline (PBS). Endogenous peroxidase was blocked using 3% hydrogen peroxide at room temperature for 25 min and washed with PBS. Protein blocking was performed with 3% bovine serum albumin at room temperature for 30 min, followed by a PBS wash. Slides were incubated with primary anti‐CD4 (Servicebio, China, GB13064, dilution 1:200) and anti‐CD8 (Servicebio, GB13068, dilution 1:200) overnight at 4°C. The next day, the slides were washed with PBS and incubated with an anti‐mouse antibody (Servicebio, GB23301, dilution 1:200) at room temperature for 50 min. After washing with PBS, the slides were treated with diaminobenzidine chromogen, and the chromogenic time was controlled under a microscope. They were then counterstained with hematoxylin stain solution for approximately 3 min, washed with tap water, differentiated with hematoxylin differentiation solution for several seconds, treated with hematoxylin returning blue solution, and washed in running water. The slides were dehydrated using an ethanol gradient and made transparent with xylene. Finally, the slides were removed from xylene and covered with cover slips.

All slides were scanned and recorded with a Pannoramic 250FLASH (3DHISTECH, Hungary) and viewed with a CaseViewer 2.4 (3DHISTECH). Computerized image analysis was performed using Alpathwell v2 (Servicebio). The staining intensities of CD4 and CD8 in single cells were classified into four categories: negative, low, moderate, and high. The immunohistopathology score (*H*‐score) was calculated using the following equation: *H*‐score = (percentage of weak‐intensity cells ×1) + (percentage of moderate‐intensity cells ×2) + (percentage of strong‐intensity cells ×3).

### 4.4 Statistical analysis

Survival analysis was performed using the Kaplan–Meier method and log rank test; univariate and multivariate Cox regression hazards models were used to evaluate survival risk factors. All factors in the univariate Cox analysis were included in multivariate Cox analysis, and the “Forward LR” method was used for multivariate Cox analysis. X‐tile was used to determine the cutoff value for the CD4 and CD8 *H*‐scores, and then transformed them into CD4+TIL and CD8+TIL low and high groups. The low and high CD4+/CD8+TIL was also determined by X‐tile.^36^ All statistical analyses were performed using SPSS 26.0 (IBM, Armonk, NY, USA) and GraphPad Prism 8.0 (GraphPad Software Inc., CA, USA). Statistical significance was set at *p* < 0.05.

## AUTHOR CONTRIBUTIONS

W. J. performed data curation, methodology, and wrote original draft. H.G., M.W., and J.L. did investigation and wrote original draft. J.Y., H.Z., and G.W. performed project administration, formal analysis, conceptualization, and reviewed and edited the manuscript. All authors have read and approved the final manuscript.

## CONFLICT OF INTEREST

The authors declare that they have no conflicts of interest.

## ETHICS STATEMENT

This study was conducted in accordance with the Declaration of Helsinki. The study protocol was reviewed and approved by the institutional review board and ethics committee of Shandong Cancer Hospital and Institute (ethical number: SDTHEC2021001025). All patients provided informed consent before paraffin‐embedded tissue sections were obtained.

## Supporting information

Figures S1‐S3

## Data Availability

The datasets used and analyzed during the current study are available from the corresponding author on reasonable request.
