# Supplementary material for: High post‐chemotherapy TIL and increased CD4+TIL are independent prognostic factors of surgically resected NSCLC following neoadjuvant chemotherapy
Source: MedComm (2020). 2023 Feb 9;4(1):e213. doi: 10.1002/mco2.213 (PMC9911612; doi:10.1002/mco2.213)
Supplement: Supplementary file 1 — Figures S1‐S3 [file MCO2-4-e213-s001.docx]

**High post-chemotherapy TIL and increased CD4+TIL are independent prognostic factors of surgical resected NSCLC following neoadjuvant chemotherapy**

**Running Head: TIL is prognostic factor of NSCLC**

Wenxiao Jia^1, 3^, Hongbo Guo^2^, Min Wang^3^, Ji Li^3^, Jinming Yu^3,*^, Hui Zhu^3,*^, Gang Wu^1,*^

^1^ Cancer Center, Union Hospital, Tongji Medical College, Huazhong University

of Science and Technology, Wuhan, 430022, China

^2^ Department of Thoracic Surgery, Shandong Cancer Hospital and Institute

affiliated to Shandong First Medical University, Shandong Academy of Medical

Sciences, 250117, Jinan, Shandong Province, China

^3^Department of Radiation Oncology, Shandong Cancer Hospital and Institute

affiliated to Shandong First Medical University, Shandong Academy of Medical

Sciences, 250117, Jinan, Shandong Province, China

**Corresponding author:** Jinming Yu, Tel: (+86)0531- 67627077; Address: 440 Jiyan Road, Jinan 250117, Shandong Province, China; E-mail: [sdyujinming@163.com](mailto:sdyujinming@163.com); Hui Zhu, Tel: (+86)0531- 67626942; Address: 440 Jiyan Road, Jinan 250117, Shandong Province, China; E-mail: [drzhuh@126.com](mailto:drzhuh@126.com); Gang Wu, Tel: (+86)027- 65655802; Address: 109 Machang Road, Wuhan 430022, Hubei Province, China; E-mail: [xhzlwg@163.com](mailto:xhzlwg@163.com).


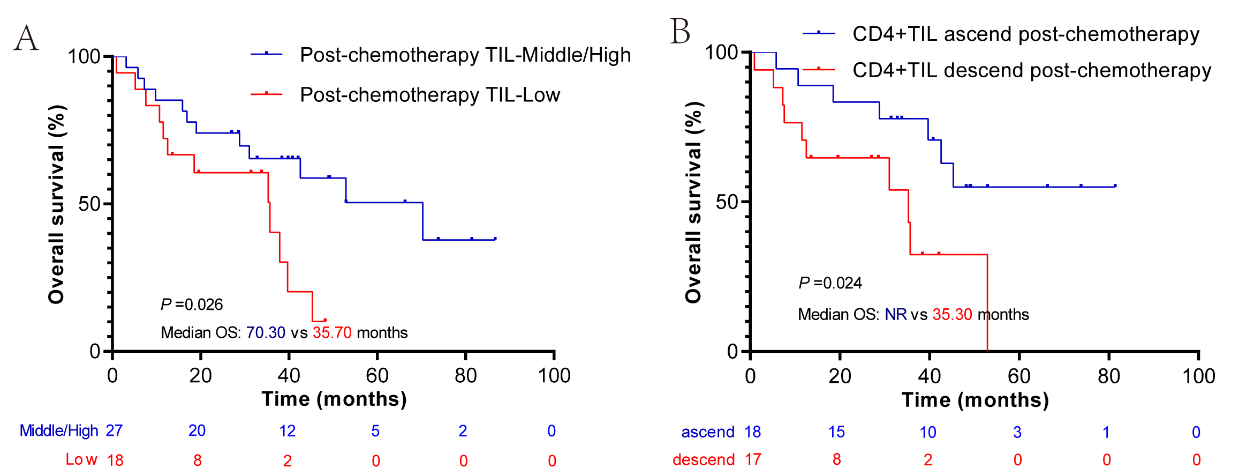


**Figure S1**：Kaplan-Meier survival analysis of post-chemotherapy TIL level and CD4+TIL ascend for NCT-NSCLC in ypTNM stage III only.


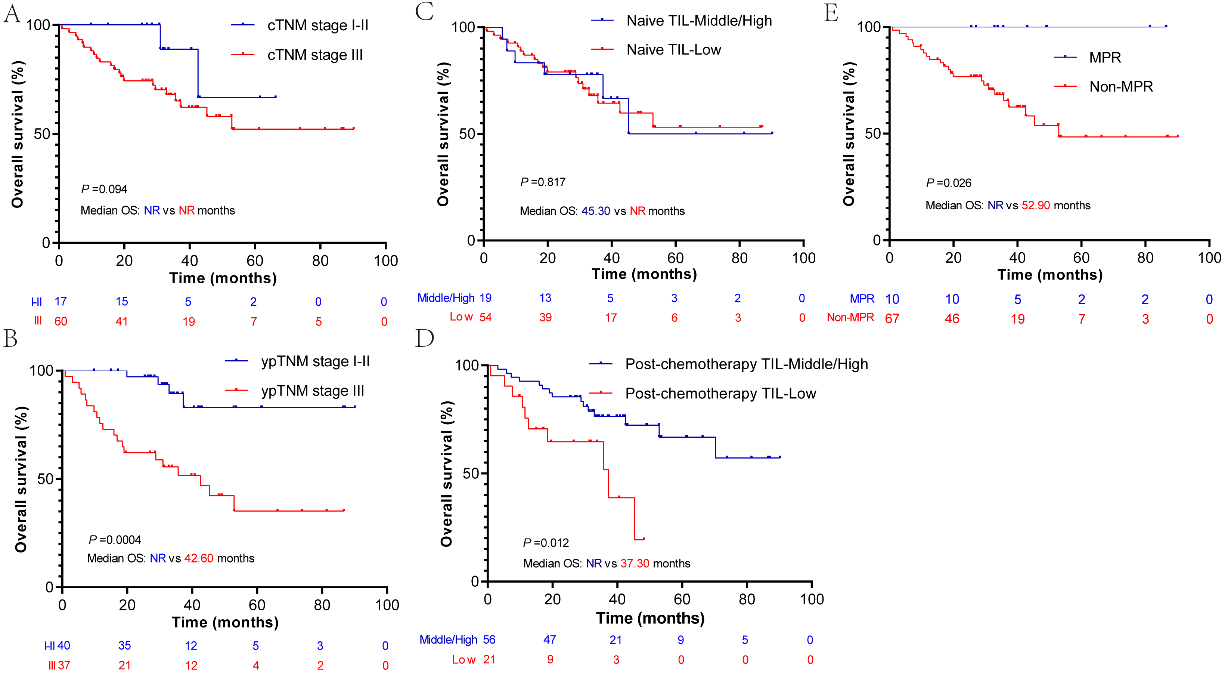


**Figure S2**: Kaplan-Meier survival analysis of cTNM, ypTNM, TIL infiltration level of naïve and post-chemotherapy tumor tissue, and MPR for NCT-NSCLC without corresponding target therapy.


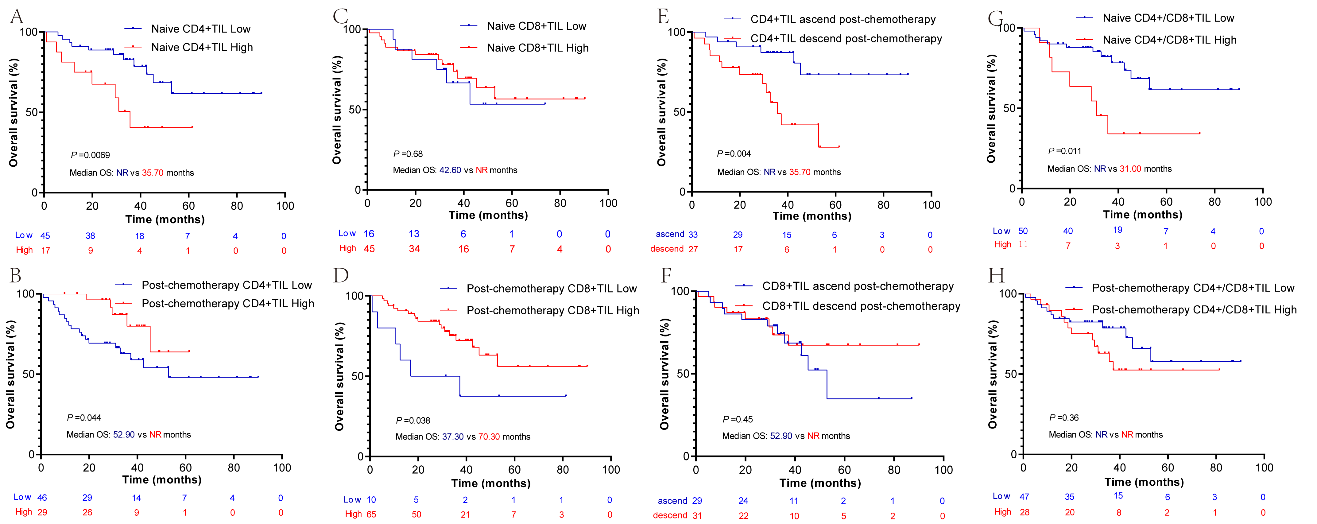


**Figure S3**: Kaplan-Meier survival analysis of CD4+TIL and CD8+TIL infiltrates, CD4+/CD8+TIL ratio in naïve and post-chemotherapy tumor tissues, CD4+TIL and CD8+TIL change post-chemotherapy for NCT-NSCLC without corresponding target therapy.
